# Single brain metastasis versus glioblastoma multiforme: a VOI-based multiparametric analysis for differential diagnosis

**DOI:** 10.1007/s11547-022-01480-x

**Published:** 2022-03-22

**Authors:** Andrea Romano, Giulia Moltoni, Alessia Guarnera, Luca Pasquini, Alberto Di Napoli, Antonio Napolitano, Maria Camilla Rossi Espagnet, Alessandro Bozzao

**Affiliations:** 1 Department of Neuroradiology, NESMOS S.Andrea Hospital, University Sapienza, Via di Grottarossa, 00135 Rome, Italy; 2 Neuroradiology Service, Department of Radiology, Memorial Sloan Kettering Cancer Center, New York, NY 10065 USA; 3 Neuroimaging lab, IRCCS Fondazione Santa Lucia, Rome, Italy; 4 Neuroradiology Unit, Imaging Department, Bambino Gesù Children’s Hospital, Rome, Italy

**Keywords:** Glioblastoma, Single brain metastasis, Perfusion, Diffusion, Differential diagnosis

## Abstract

**Purpose:**

The authors’ purpose was to create a valid multiparametric MRI model for the differential diagnosis between glioblastoma and solitary brain metastasis.

**Materials and methods:**

Forty-one patients (twenty glioblastomas and twenty-one brain metastases) were retrospectively evaluated. MRIs were analyzed with Olea Sphere^®^ 3.0. Lesions’ volumes of interest (VOIs) were drawn on enhanced 3D T1 MP-RAGE and projected on ADC and rCBV co-registered maps. Another two VOIs were drawn in the region of hyperintense cerebral edema, surrounding the lesion, respectively, within 5 mm around the enhancing tumor and into residual edema. Perfusion curves were obtained, and the value of signal recovery (SR) was reported. A two-sample *T* test was obtained to compare all parameters of GB and BM groups. Receiver operating characteristics (ROC) analysis was performed.

**Results:**

According to ROC analysis, the area under the curve was 88%, 78% and 74%, respectively, for mean ADC VOI values of the solid component, the mean and max rCBV values in the perilesional edema and the PSR. The cumulative ROC curve of these parameters reached an area under the curve of 95%. Using perilesional max rCBV > 1.37, PSR > 75% and mean lesional ADC < 1 × 10^−3^ mm^2^ s^−1^ GB could be differentiated from solitary BM (sensitivity and specificity of 95% and 86%).

**Conclusion:**

Lower values of ADC in the enhancing tumor, a higher percentage of SR in perfusion curves and higher values of rCBV in the peritumoral edema closed to the lesion are strongly indicative of GB than solitary BM.

## Introduction

Glioblastoma (GB) and brain metastases (BM) are the two most common malignant tumors of the central nervous system in adults. A known primary neoplasm history and the presence of multiple brain lesions are the two main data leading to the diagnosis of metastasis. However, around 40–50% of BM begin with a solitary lesion, and BM can be the first manifestation of a still unknown extracerebral malignancy [1]. Moreover, GB can occur in patients with systemic cancer [2]. On conventional magnetic resonance imaging (MRI), these two lesions may appear very similar, mostly characterized by central necrosis, inhomogeneous ring enhancement and surrounded by edema. Thereby, the radiological differential diagnosis may result challenging, even if it is extremely important in terms of patient management and prognosis [3]. Although conventional magnetic resonance imaging is similar, there are significant histopathological differences between GB and BM. The capillaries of brain metastasis lack blood–brain barrier (BBB), as well as those from the site of the original systemic cancer. This capillary ultrastructure results in a greatly increased capillary permeability that, together with the expansive growth of metastases, is responsible for peripheral vasogenic edema. On the other hand, GB is characterized by a high rate of neoangiogenesis with capillary ultrastructure similar to the one of the normal brain [4]. Furthermore, histopathological examinations in GB showed the presence of tumor cells scattered in the peritumoral area [5]. Therefore, peritumoral edema in GB is better referred to as infiltrative edema.

Morphometric parameters and MR signal characteristics of the tumoral mass and peritumoral area evaluation were proposed to distinguish GB and BM [6]; the authors assessed that GB is recognizable from a lower ratio of the maximum diameter of the peritumoral area measured on T2-weighted images to the maximum diameter of the enhancing mass area.

The role of advanced MRI techniques including spectroscopy, perfusion imaging, diffusion tensor imaging and the measurement of the apparent diffusion coefficient (ADC) has been investigated in order to reach a radiological differential diagnosis between GB and BM [3, 4, 7–14]. Both the enhanced area and the peritumoral area have been analyzed, the second with more consistent results [3, 4, 15, 16]. ADC and dynamic susceptibility-weighted contrast-enhanced (DSC) perfusion MRI seem to be very promising imaging tools to differentiate GB from BM [3, 4, 7, 15, 16]. The former because is related to lesions cellularity, the latter because provides information both on neoangiogenesis by rCBV value’s analysis and on capillary permeability by hemodynamic curve’s analysis.

The authors’ purpose was to evaluate ADC and rCBV values in the enhanced lesion, in the peritumoral area and in distal edema using a volume of interest (VOI) based method and to analyze hemodynamic curves obtained from DSC perfusion MRI, in order to create a valid multiparametric MRI model for the differential diagnosis between GB and solitary BM.

## Methods

### Study population

Institutional review board approval was obtained for the realization of this retrospective study. Written informed consent for MR examination was acquired from every patient.

Patients were retrospectively recruited by reviewing the imaging archive of our institution from 2010 to 2019 with the following inclusion criteria: evidence of GB or single BM confirmed by histological examination; MRI examination including cerebral examination by means of FLAIR, DWI, 3D T1 MP-RAGE and DSC perfusion images. Patients affected by severe comorbidities, previous surgery or trauma and whose MRI exams were discarded by artifacts that were considered ineligible.

Images were evaluated by a 5-year-experienced neuroradiology fellow (A.G.), who was blinded to clinical data.

The final cohorts consisted of 20 patients affected by GB and 21 patients affected by single BM. Metastases were secondary to lung cancer in 11 patients; colon-rectal cancer in 3 patients; melanoma in 2 patients; and hepatic cell carcinoma, endometrial cancer, pancreatic adenocarcinoma, renal cell carcinoma and neuroendocrine tumor in 1 patient, respectively.

### MRI protocol

MRI images were acquired before surgery, radiotherapy and chemotherapy. All subjects were examined using a Siemens (Siemens, Enlargen, Germany) Magnetom Sonata scanner (1.5 T). The imaging protocol included high-resolution 3D T1WI MP-RAGE sequence before and after intravenous administration of paramagnetic contrast agent (0.1 mmol/kg), as anatomical reference; T2WI and FLAIR to exclude other cerebral pathologies. Before the contrast injection, DWI was obtained in the axial plane using echo-planar sequence with the following parameters: the *b* values were 0, 500 and 1000 mm^2^/s. ADC maps images were generated automatically by the MRI unit.

DSC perfusion images were obtained with a T2*WI GRE EPI (TR/TE 1490/40 ms; flip angle 90°; FOV 230 × 230 mm; matrix 128 × 128, 14 sections of 5 mm thickness; acquisition time 78 s) during gadopentetate dimeglumine (DOTAREM^®^; dose 0.1 mmol/kg, injection rate 4 ml/s) bolus injection, followed by a saline flush of 20 ml. Fifty measurements were acquired, allowing at least five measurements before bolus arrival.

### Imaging analysis

MRIs were analyzed with Olea Sphere^®^ 3.0 (Olea Medical, La Ciotat, France), in particular with diffusion, perfusion and volume of interest segmentation plug-ins. The arterial input function was selected automatically, and multiparametric perfusion maps were calculated using block-circulant singular value decomposition technique for DSC. The rCBV maps derived from DSC perfusion datasets were then exported from the software for subsequent analysis.

FLAIR, 3D T1 MP-RAGE images, ADC and rCBV maps for each patient were co-registered by the OleaSphere software; this was followed by visual inspection to ensure adequate alignment.

**Fig. 1:**
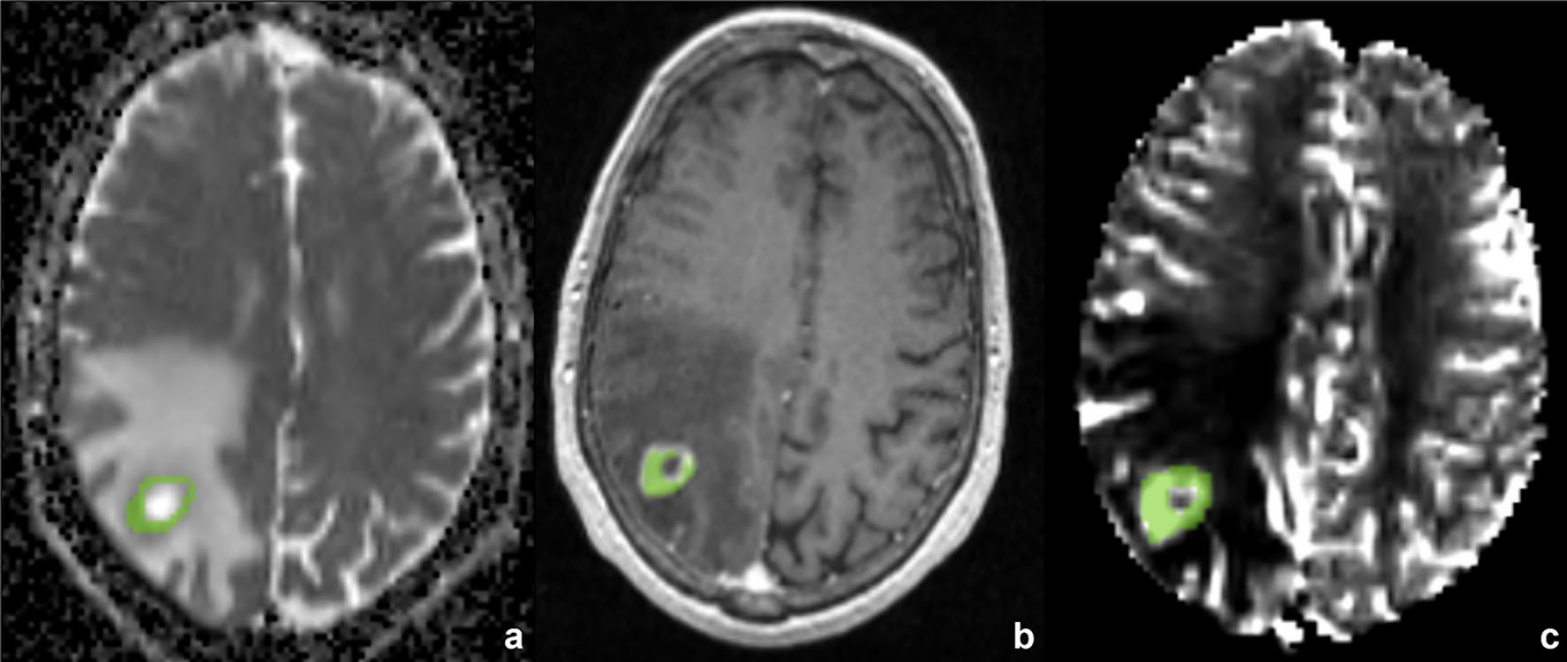
Volumes of interest (VOIs) of the lesions were drawn on enhanced 3D T1 MP-RAGE avoiding cyst or necrotic degeneration and projected on ADC and rCBV co-registered maps

First of all, volumes of interest (VOIs) of the lesions were drawn on enhanced 3D T1 MP-RAGE avoiding cyst or necrotic degeneration and then projected on ADC and rCBV co-registered maps (Fig. 1). Data recorded included minimum and medium values obtained from VOIs, respectively, in the ADC maps and in the corrected rCBV maps.

Another 2 VOIs were drawn in the region of hyperintense cerebral edema, surrounding the lesion (GB or BM) visible on FLAIR images. The first VOI was drawn into perilesional edema within 5 mm around the enhancing tumor. The second VOI was drawn into residual edema. Both VOIs were projected on ADC and rCBV maps. Data recorded included minimum and medium values obtained from VOIs, respectively, in the ADC maps and in the corrected rCBV maps.

Perfusion curves were obtained for each lesion, and the value of signal recovery (SR) was reported. PSR (percent signal recovery) was calculated with the formula: PSR = 100−(100 * SR)/PH inside each VOI of the solid component of the brain lesion.

### Statistical analysis

The statistical evaluation was performed with SPSS software, version 20.0, Chicago, IL, USA.

A two-sample *T* test was obtained to compare all parameters (lesional and peritumoral rCBV, ADC and lesional PSR) of GB and BM groups.

Receiver operating characteristics (ROC) analysis was performed to determine the optimal parameter in distinguishing GB from BM. Optimal thresholds were calculated for each ROC curve to maximize both sensitivity and specificity. Subsequently, a combined ROC curve for combination of parameters was calculated extrapolating from the maximum likelihood estimation model of combining classifiers. The area under the curve was calculated for each individual classifier’s ROC curve as well as for the combined ROC curves.

## Results

All values obtained from the analyzed parameters showed a Gaussian distribution. Comparing all parameters evaluated for patients with GB and BM, the cerebral lesions were distinguishable with the mean ADC VOI values of solid component (BM: 1.13 × 10^–3^ mm^2^/s, GB: 0.7 × 10^–3^ mm^2^/s; *p* < 0.001), the PSR values (BM: 71%, GB: 84%; *p* = 0.003) and the mean and max rCBV values in the perilesional edema within 5 mm around the enhancing tumor (mean rCBV values BM: 0.87, GB: 1.46; *p* = 0.026), (max rCBV values BM: 1.21, GB: 2.63; *p* = 0.001). rCBV values of the solid lesions and ADC VOI values of the perilesional edema did not show a statistically significant difference between BM and GB (Table 1).

**Table 1:** Comparison between single brain metastasis and glioblastoma according to multiparametric diffusion and DSC perfusion evaluation

		Metastasis	Glioblastoma	*p*
		Mean values (standard deviation)		
*Perilesional edema*	ADC* min	1.28 (0.29)	1.25 (0.28)	NS
	ADC*mean	1.50 (0.24)	1.43 (0.31)	NS
	rCBV max	1.21 (0.64)	2.63 (1.72)	**0.002**
	rCBV mean	0.87 (0.22)	1.46 (1.08)	**0.026**
*Edema*	ADC* min	1.44 (0.34)	1.36 (0.34)	NS
	ADC*mean	1.57 (0.30)	1.52 (0.36)	NS
	rCBV max	0.97 (0.90)	1.67 (1.54)	NS
	rCBV mean	0.61 (0.56)	0.88 (0.70)	NS
*Lesion*	ADC* min	0.56 (0.28)	0.43 (0.20)	NS
	ADC*mean	1.13 (0.21)	0.71 (0.33)	**0.0001**
	rCBV mean	9.71 (3.53)	7.60 (4.12)	NS
	rCBV max	71.14 (13..84)	84.59 (13.30)	NS
*PSR*		4.62 (2.78)	4.89 (2.88)	**0.003**

^*^ADC values should be multiplied by 10^−3^ and expressed in units of mm^2^/s. In bold statistically significant results (*p* value ≤ 0.05)

**Fig. 2:**
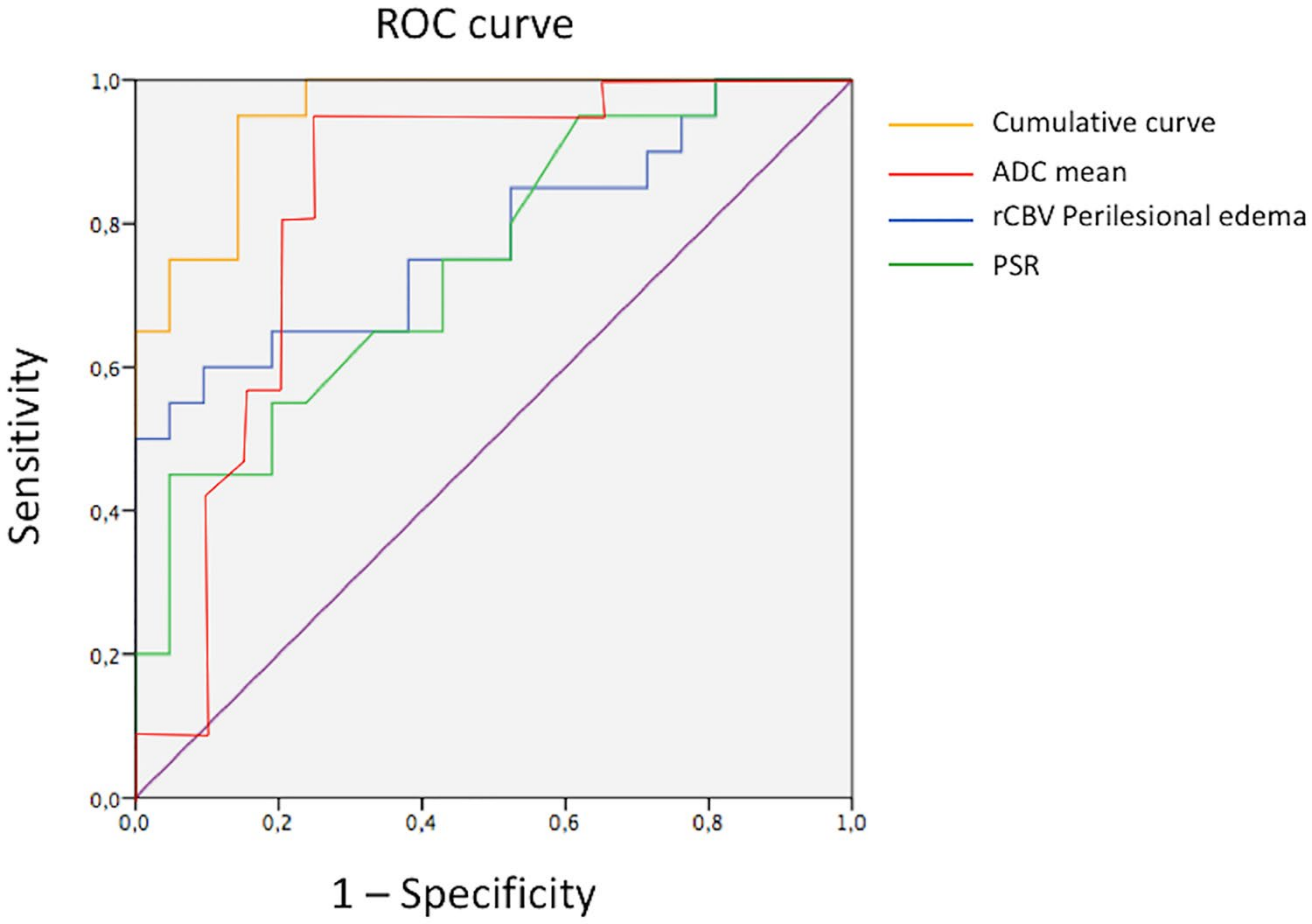
According to ROC analysis, the area under the curve was 88%, 78% and 74%, respectively, for mean ADC VOI values of the solid component, the mean and max rCBV values in the perilesional edema and the PSR. The cumulative ROC curve of these parameters reached an area under the curve of 95%

According to ROC analysis, the area under the curve was 88%, 78% and 74%, respectively, for mean ADC VOI values of the solid component, the mean and max rCBV values in the perilesional edema and the PSR. The combined ROC curve of these parameters reached an area under the curve of 95% (Fig. 2).

Values of max rCBV = 1.37 in the perilesional edema, PSR = 75% and mean lesional ADC = 1 × 10^–3^ mm^2^/s represented an optimal cutoff point for distinguishing subjects with BM or GB perilesional max rCBV sensitivity: 75%, specificity: 62%; PSR sensitivity: 70%, specificity: 58%; lesional mean ADC sensitivity: 81%, specificity: 75%).

Using perilesional max rCBV > 1.37, PSR > 75% and mean lesional ADC < 1 × 10^–3^ mm^2^/s GB could be differentiated from solitary BM with sensitivity and specificity of 95% and 86%.

## Discussion

Our study showed that it is possible to distinguish a GB from a solitary BM using a multiparametric analysis and a VOI-based method, with a sensitivity of 95% and a specificity of 86%.

The differentiation of metastasis from other malignant tumors on conventional MRI is usually straightforward due to the clinical history of the patient or the existence of multiple lesions [17]. We know that the differentiation of glioma from single brain metastasis is clinically crucial, because it affects the clinical outcome of patients and changes patient management. As GB and BM have similar conventional MRI characteristics, advanced MRI techniques can be useful to evaluate some features of the tumor, such as cellularity, ultrastructure of tumor capillaries and permeability that differ greatly between GB and BM [3, 4, 18]. Furthermore, it has been found that glioma tends to infiltrate the peritumoral edema region as well, while this condition is not typical of brain metastases [18].

Previously, some studies tried to evaluate the role of MRI with a single or multimodal approach for differentiating glioma from brain metastasis [3, 4, 10–25]. More frequently, diffusion MRI and DSC perfusion techniques were applied, alone or in association with MR spectroscopy and diffusion tensor imaging. Usually, a ROI-based analysis was utilized; only Qin et al. [10] used a VOI-based method for a histogram analysis concerning the perfusion DSC technique. We use a VOI-based method to verify diffusion and perfusion differences between GB and solitary BM in both solid tumor portion and peritumoral edema.

By using the VOIs to include only the solid component of the tumor tissue, avoiding cyst and necrotic degeneration, the results are more reliable and allow a better and more objective evaluation of images than using a ROI-based method. Indeed, results of the VOI method, as well as the ones of the histogram method, showed greater interobserver agreement and diagnostic accuracy than the localized hotspot ROI method [10].

In contrast with almost all previous studies, we found a statistically significant difference (*p* < 0.001) between the mean ADC VOI values in the solid portion of the tumors, lower in GB than in BM. Reviewing the literature, there is not agreement in distinguishing these lesions by using ADC values. Lee et al. and Tsougos et al. imputed the absence of significant differences among the ADC values of these lesions to the heterogeneous signal intensity due to necrosis and susceptibility artifacts [13, 16]. Only Chiang et al. reported similar results to ours, assessing that the higher ADC in metastasis suggests higher intracellular and extracellular water fractions than in high-grade gliomas [25]. More recently, Poulon et al. [26] compared specimens of twenty-five patients with brain tumors including GB and BM. They reported that brain metastases were characterized by hypercellularity and disorganized stroma with numerous blood vessels and dense collagen network. On the other hand, in GB samples the solid tumor component was associated with a highly disorganized tumor cell architecture with microvascular proliferation. Since it is well known that that brain neoplasm with higher cellularity showed a significant reduction in ADC values, the structural pattern showed by Poulon et al. could explain ours and Chiang et al. results [25, 26] characterized by higher ADC values in BM as an expression of the vascular and collagen components than in GB characterized by higher cellularity.

We believe that in our study the substantial results about ADC values are also related to the use of VOIs that include only the solid component of tumoral tissue, avoiding cyst or necrotic degeneration, instead of using the ROI-based method.

According to the literature, we confirm that it is not possible to distinguish solitary BM and GB using the rCBV values of perfusion MRI in the solid portion of the tumor, also using the VOI-based method. rCBV as a biomarker of increased angiogenesis should therefore be interpreted with caution in differentiating BM and GB, particularly within enhancing tumor [3]. Only Qin et al. [10] in a recent study, using a histogram analysis, reported different results with GBMs characterized by higher perfusion and more heterogeneous status in the distribution of blood perfusion due to a lower different expression level of vascular epidermal growth factor, than to metastasis.

Nevertheless, the evaluation of the percent value of signal recovery (PSR) derived from the Δ*R*^2^* curve of DSC perfusion MR imaging is a method that can distinguish these lesions. We obtained a significant difference (*p* = 0.003) between GB and BM with lower recovery of signal intensity inside the lesion for metastasis group. Cha et al. [4] assessed that the significant difference in the percentage of signal intensity recovery between GB and BM, reduced in metastasis, is probably due to the difference in capillary permeability. Capillaries of metastatic brain tumor in fact resemble those of systemic origin and are associated with a defective endothelium, devoid of any rudimentary BBB architecture. The same results were obtained from Neska-Matuszewska et al. [11]. Different results were discussed in recent article of Lee et al. [27]. The authors reported no significant differences in the PSR between BM and GB. They assessed that the contrast agent pre-load administration and pulse sequence parameters eliminated these PSR differences. In our cohort, the lower sensitivity and specificity results, despite the significant difference found between the two groups, are likely due to the greater number of patients with brain metastasis from lung cancer, lesions with less vascularization compared to others (for example metastasis from kidney or melanoma) [18]. We suppose that the lower representation of pathological capillaries could justify the reduced permeability of these lesions and the partial signal recovery compared to GB.

The highly aggressive nature of GB is associated with their infiltrative growth in the peritumoral area exceeding the limits of the enhancing tumor core, while metastases usually grow by expansion, displacing the surrounding brain tissue, which shows pure vasogenic edema [11]. In GB, the peritumoral brain zone has already been evaluated in the literature using DWI or PWI, showing increased values of rCBV accepted by all, and controversial results in the ADC values [3, 11, 13–16, 20]

Indeed, the peritumoral rCBV derived from DSC represents a valid parameter to distinguish metastasis and GB with higher values of rCBV in GB peritumoral edema due to an infiltrative process, which is not found in solitary BM [11, 13, 15, 17, 18, 28]. In brain metastases vasogenic edema associated with the leakage through abnormal capillary walls allows compression of the microcirculation close to the lesion and reduction of rCBV values [14].

In our study, this result is evident only in VOIs drawn within 5 mm around the enhancing tumor; on the other hand, in regions located far from this spatial limit, only a trend of significant results was appreciable suggesting that in GB peritumoral zone there is a decreasing gradient of rCBV values from the area close to the enhancing solid lesion to the normal white matter, while in BM the rCBV values in these regions were similar without any gradient appearance. This interesting result is in accord with the study of She et al. [15] reflecting the gradient of infiltrative pattern of GB.

Concerning the peritumoral zone, ADC represents a parameter with debated results in the literature. Some authors assessed the role of diffusion biomarkers in recognizing the presence of tumoral infiltration in GB [3, 11, 16, 18]. The significantly increased ADC value in edema surrounding metastases suggests that they cause more fluid production than high-grade gliomas [18]. Lee et al. [16] found a significantly lower minimum ADC value in the peritumoral edema of GB than of BM, identifying infiltrative peritumoral edema in GB.

Some authors [13] did not find any difference comparing ADC values in GB and BM peritumoral edema, as well as us. Indeed, we reported that mean and minimum ADC VOI values neither in peritumoral edema nor in distant edema are useful to differentiate infiltrative edema in GB from vasogenic edema in metastasis.

We cannot exclude that a ROI-based analysis used in previous studies is associated with lower accuracy than a VOI-based method. On the other hand, our analysis was limited to ADC values and did not include other diffusion biomarkers such as mean diffusivity (MD) and fractional anisotropy (FA).

The strength of our results is highlighted by the combined ROC curve analysis where the rule and the power of multiparametric evaluation of tumoral cerebral disease leads to obtain good results in differentiating single cerebral metastasis vs GB. The choice of using combined ROC curve reflected the importance of a multiparametric analysis.

Our study has several limitations: the retrospective nature of the analysis limits the generalizability of the results. Another limit is the patient sample size; this could represent a potential bias in our results. Despite this, we are the first that applied a VOI evaluation of these lesions, a method, as reported previously, that allow more reliable results and objective evaluation than using a ROI-based method, applied in most of papers in literature. Moreover, we focused on MRI parameters, but there is a lack of histopathologic correlation between imaging parameters and surgical specimens, and its results should be validated in prospective studies with strict histopathologic, although such point-to-point correlations are very difficult to obtain. Texture analysis and artificial intelligence could be an alternative method to distinguish GB from MB; as reported in some recent articles, peri-enhancing edema [29–31] and enhancing lesions [31, 32] represent the target of research, with promising results.

## Conclusions

The combination of multiple parameters allows for increased diagnostic power.

This approach confirmed that the differential diagnosis between GB and solitary BM is related to several parameters depending on different tumoral components and on the lesion growth pattern, infiltrative versus expansive.

The global evaluation of these parameters leads more easily to a correct diagnosis, with an AUC of 0.95, higher than any other individual parameter.

We can conclude that lower values of ADC in the enhancing tumor volume and a higher percentage of signal recovery in perfusion curves, associated with higher values of rCBV in the peritumoral edema closed to the lesion, are strongly indicative of GB than solitary BM.

## Declarations

### Data availability

The datasets generated during and/or analyzed during the current study are available from the corresponding author on reasonable request**.**

## References

[1] Nayak L, Lee EQ, Wen PY (2012). Epidemiology of brain metastases. Curr Oncol Rep.

[2] Piccirilli M, Salvati M, Bistazzoni S, Frati A, Brogna C, Giangaspero F, Frati R, Santoro A (2018). Glioblastoma multiforme and breast cancer: report on 11 cases and clinico-pathological remarks. Tumori.

[3] Bauer AH, Erly W, Moser FG (2015). Differentiation of solitary brain metastasis from glioblastoma multiforme: a predictive multiparametric approach using combined MR diffusion and perfusion. Neuroradiology.

[4] Cha S, Lupo JM, Chen MH (2007). Differentiation of glioblastoma multiforme and single brain metastasis by peak height and percentage of signal intensity recovery derived from dynamic susceptibility-weighted contrast-enhanced perfusion MR imaging. Am J Neuroradiol.

[5] D’Alessio A, Proietti G, Sica G, Scicchitano BM (2019). Pathological and molecular features of glioblastoma and its peritumoral tissue. Cancers (Basel).

[6] Maurer MH, Synowitz M, Badakshi H, Lohkamp LN, Wüstefeld J, Schäfer ML, Wiener E (2013). Glioblastoma multiforme versus solitary supratentorial brain metastasis: differentiation based on morphology and magnetic resonance signal characteristics. Rofo.

[7] Tsolaki E, Svolos P, Kousi E, Kapsalaki E, Fountas K, Theodorou K, Tsougos I (2013). Automated differentiation of glioblastomas from intracranial metastases using 3T MR spectroscopic and perfusion data. Int J Comput Assist Radiol Surg.

[8] Mouthuy N, Cosnard G, Abarca-Quinones J, Michoux N (2012). Multiparametric magnetic resonance imaging to differentiate high-grade gliomas and brain metastases. J Neuroradiol.

[9] Ishimaru H, Morikawa M, Iwanaga S, Kaminogo M, Ochi M, Hayashi K (2001). Differentiation between high-grade glioma and metastatic brain tumor using single-voxel proton MR spectroscopy. Eur Radiol.

[10] Qin J, Li Y, Liang D (2019). Histogram analysis of absolute cerebral blood volume map can distinguish glioblastoma from solitary brain metastasis. Medicine.

[11] Neska-Matuszewska M, Bladowska J, Sąsiadek M, Zimny A (2018). Differentiation of glioblastoma multiforme, metastases and primary central nervous system lymphomas using multiparametric perfusion and diffusion MR imaging of a tumor core and a peritumoral zone—Searching for a practical approach. PLoS ONE.

[12] Calli C, Kitis O, Yunten N (2006). Perfusion and diffusion MR imaging in enhancing malignant cerebral tumors. Eur J Radiol.

[13] Tsougos I, Svolos P, Kousi E (2012). Differentiation of glioblastoma multiforme from metastatic brain tumor using proton magnetic resonance spectroscopy, diffusion and perfusion metrics at 3 T. Cancer Imaging.

[14] Law M, Cha S, Knopp EA (2002). High-grade gliomas and solitary metastases: differentiation by using perfusion and proton spectroscopic MR imaging. Radiology.

[15] She D, Xing Z, Cao D (2019). Differentiation of glioblastoma and solitary brain metastasis by gradient of relative cerebral blood volume in the peritumoral brain zone derived from dynamic susceptibility contrast perfusion magnetic resonance imaging. J Comput Assist Tomogr.

[16] Lee EJ, TerBrugge K, Mikulis D (2011). Diagnostic value of peritumoral minimum apparent diffusion coefficient for differentiation of glioblastoma multiforme from solitary metastatic lesions. AJR Am J Roentgenol.

[17] Svolos P, Tsolaki E, Kapsalaki E, Theodorou K, Fountas K, Fezoulidis I, Tsougos I (2013). Investigating brain tumor differentiation with diffusion and perfusion metrics at 3T MRI using pattern recognition techniques. Magn Reson Imaging.

[18] Suh CH, Kim HS, Jung SC, Choi CG, Kim SJ (2018). Perfusion MRI as a diagnostic biomarker for differentiating glioma from brain metastasis: a systematic review and meta-analysis. Eur Radiol.

[19] Jung SC, Choi SH, Yeom JA, Kim JH, Ryoo I, Kim SC, Shin H, Lee AL, Yun TJ, Park CK, Sohn CH, Park SH (2013). Cerebral blood volume analysis in glioblastomas using dynamic susceptibility contrast-enhanced perfusion MRI: a comparison of manual and semiautomatic segmentation methods. PLoS ONE.

[20] Lee EJ, Ahn KJ, Lee EK, Lee YS, Kim DB (2013). Potential role of advanced MRI techniques for the peritumoural region in differentiating glioblastoma multiforme and solitary metastatic lesions. Clin Radiol.

[21] Li X, Wang D, Liao S, Guo L, Xiao X, Liu X, Xu Y, Hua J, Pillai JJ, Wu Y (2020). Discrimination between glioblastoma and solitary brain metastasis: comparison of inflow-based vascular-space-occupancy and dynamic susceptibility contrast MR imaging. AJNR Am J Neuroradiol.

[22] Server A, Orheim TE, Graff BA, Josefsen R, Kumar T, Nakstad PH (2011). Diagnostic examination performance by using microvascular leakage, cerebral blood volume, and blood flow derived from 3-T dynamic susceptibility-weighted contrast-enhanced perfusion MR imaging in the differentiation of glioblastoma multiforme and brain metastasis. Neuroradiology.

[23] Sunwoo L, Yun TJ, You SH, Yoo RE, Kang KM, Choi SH, Kim JH, Sohn CH, Park SW, Jung C, Park CK (2016). Differentiation of glioblastoma from brain metastasis: qualitative and quantitative analysis using arterial spin labeling MR imaging. PLoS ONE.

[24] Wang S, Kim S, Chawla S, Wolf RL, Zhang WG, O'Rourke DM, Judy KD, Melhem ER, Poptani H (2009). Differentiation between glioblastomas and solitary brain metastases using diffusion tensor imaging. Neuroimage.

[25] Chiang IC, Kuo YT, Lu CY, Yeung KW, Lin WC, Sheu FO, Liu GC (2004). Distinction between high-grade gliomas and solitary metastases using peritumoral 3-T magnetic resonance spectroscopy, diffusion, and perfusion imagings. Neuroradiology.

[26] Poulon F, Pallud J, Varlet P, Zanello M, Chretien F, Dezamis E, Abi-Lahoud G, Nataf F, Turak B, Devaux B, Abi HD (2018). Real-time Brain Tumor imaging with endogenous fluorophores: a diagnosis proof-of-concept study on fresh human samples. Sci Rep.

[27] Lee MD, Baird GL, Bell LC, Quarles CC, Boxerman JL (2019). Utility of percentage signal recovery and baseline signal in DSC-MRI optimized for relative CBV measurement for differentiating glioblastoma, lymphoma, metastasis, and meningioma. AJNR Am J Neuroradiol.

[28] Lehmann P, Saliou G, de Marco G, Monet P, Souraya SE, Bruniau A, Vallée JN, Ducreux D (2012). Cerebral peritumoral oedema study: does a single dynamic MR sequence assessing perfusion and permeability can help to differentiate glioblastoma from metastasis?. Eur J Radiol.

[29] Dong F, Li Q, Jiang B, Zhu X, Zeng Q, Huang P, Chen S, Zhang M (2020). Differentiation of supratentorial single brain metastasis and glioblastoma by using peri-enhancing oedema region-derived radiomic features and multiple classifiers. Eur Radiol.

[30] Skogen K, Schulz A, Helseth E, Ganeshan B, Dormagen JB, Server A (2019). Texture analysis on diffusion tensor imaging: discriminating glioblastoma from single brain metastasis. Acta Radiol.

[31] Bae S, An C, Ahn SS (2020). Robust performance of deep learning for distinguishing glioblastoma from single brain metastasis using radiomic features: model development and validation. Sci Rep.

[32] Zhang G, Chen X, Zhang S, Ruan X, Gao C, Liu Z, Wei X (2019). Discrimination between solitary brain metastasis and glioblastoma multiforme by using ADC-based texture analysis: a comparison of two different ROI placements. Acad Radiol.

